# On some invariants of cubic fourfolds

**DOI:** 10.1007/s40879-023-00651-y

**Published:** 2023-07-11

**Authors:** Frank Gounelas, Alexis Kouvidakis

**Affiliations:** 1 Fakultät für Mathematik und Informatik, Georg-August-Universität Göttingen, Bunsenstr. 3-5, 37073 Göttingen, Germany; 2 Department of Mathematics and Applied Mathematics, University of Crete, 70013 Heraklion, Greece

**Keywords:** Cubic fourfold, Fano scheme, Second type locus, Invariants, 14J70, 14J35, 14M15

## Abstract

For a general cubic fourfold $X\subset \mathbb {P}^5$ with Fano variety *F*, we compute the Hodge numbers of the locus $S\subset F$ of lines of second type and the class of the locus $V\subset F$ of triple lines, using the description of the latter in terms of flag varieties. We also give an upper bound of 6 for the degree of irrationality of the Fano scheme of lines of any smooth cubic hypersurface.

## Introduction

Let $X\subset \mathbb {P}^5_\textbf{C}$ be a general cubic fourfold and $F=F(X)\subset \textrm{G}(2,6)$ its Fano scheme of lines, which is a four-dimensional hyperkähler variety. The normal bundle of a line $\ell \in F$ decomposes as one of the following two:and $\ell$ is called of first or second type respectively. The locus of second type lines is a smooth projective irreducible surface $S\subset F$ which has drawn considerable interest since the landmark paper [5] of Clemens–Griffiths. The aim of this paper is to study some invariants of *S* and *F*.

In Sect. 2 we summarise what is known about *S* and *F* and fix notation. In Sect. 4 we use Amerik’s description of the second type locus *S* as the degeneracy locus of the universal Gauss map

$$\begin{aligned}&f:{\text {Sym}}^2\mathscr {U}_F\rightarrow \mathscr {Q}^\vee _F\\&\quad S=D_2(f)\subset F, \end{aligned}$$

the Harris–Tu formula as well as Borel–Bott–Weil computations on the Grassmannian from Sect. 3 to compute the Hodge numbers of *S*.

### Theorem A

If $X\subset \mathbb {P}^5$ is a general cubic then the second type locus $S\subset F$ is a smooth irreducible surface whose Hodge numbers are as follows:

$$\begin{aligned} h^{1,0}&=q=0, \\ h^{2,0}&=p_g=449, \\ h^{1,1}&=1665, \end{aligned}$$

whereas $\pi _1(S,s)$ contains a non-trivial element of order 2.

The order of the torsion element in the above theorem was pointed out to us by Huybrechts (see Remark 4.4), who also independently calculated the above invariants in his lecture notes on cubic hypersurfaces, although our approach using Borel–Bott–Weil directly on *F* leads to a more refined analysis of the projective embedding *S* in the Plücker space.

In the final Sect. 6 we extend results from [11] to prove the following

### Theorem B

Let $X\subset \mathbb {P}^{\,n+1}$ be a smooth cubic hypersurface and *F*(*X*) its Fano scheme of lines. Then degree of irrationality of *F*(*X*), i.e., the minimal degree of a dominant, generically finite, rational map to $\mathbb {P}^{2(n-2)}$, satisfies

$$\begin{aligned} \textrm{irr}\hspace{0.55542pt}(F(X))\leqslant 6. \end{aligned}$$

## Background and notation

As the notation surrounding cubic fourfolds is substantial, we devote this section to fixing that used in the paper and recalling some basic properties, so that it acts as a reference for later sections.

For a vector bundle *E* we denote by , so that projective space parametrises one-dimensional subspaces. We denote by $\textrm{G}(k,n)$ the space of *k*-dimensional subspaces of $\textbf{C}^n$, with universal bundle $\mathscr {U}$ of rank *k* and universal quotient bundle $\mathscr {Q}$ of rank $n-k$. We will denote by $\sigma _I$ the standard Schubert cycles for an index *I* so that, e.g., $\sigma _i={\mathrm c}_i(\mathscr {Q})$ for $i\geqslant 1$.

Throughout, $X\subset \mathbb {P}^5$ will be a smooth cubic fourfold with $H_X=\mathscr {O}_{X}(1)$ and $F\subset \textrm{G}(2,6)$ the Fano scheme of lines contained in *X* which is a hyperkähler fourfold [3]. To unburden notation, we will often be sloppy in distinguishing a line $\ell \subset X$ and the point $[\ell ]\in F$ that it defines. We denote by $\mathscr {U}_F,\mathscr {Q}_F$ the restrictions of $\mathscr {U},\mathscr {Q}$ to *F*.

The subvariety $F\subset \textrm{G}(2,6)$ is given by a section of the rank four bundle${\text {Sym}}^3\mathscr {U}^*\cong q_*p^*\mathscr {O}_{\mathbb {P}^5}(3)$ where *p*, *q* are the projections from the universal family $\textrm{G}(2,6)\leftarrow I\rightarrow \mathbb {P}^5$. In fact it is the section induced, under this isomorphism, by $f\in k[x_0,\ldots ,x_5]_3$ whose vanishing is *X* (see [6, Proposition 6.4]) and its cohomology class in the Grassmannian is given by ${\mathrm c}_4({\text {Sym}}^3\mathscr {U}^*)$ which can be computed as follows (see [9, Example 14.7.13]):

1 $$\begin{aligned} \begin{aligned} \left[ F \right]&= 18{\mathrm c}_1(\mathscr {U}^*)^2{\mathrm c}_2(\mathscr {U}^*) + 9{\mathrm c}_2(\mathscr {U}^*)^2 \\&= 18\sigma _1^2\sigma _{1,1}+9\sigma _{1,1}^2 = 27\sigma _2^2-9\sigma _1\sigma _3-18\sigma _4. \end{aligned} \end{aligned}$$

Following [5], there are two types of lines $\ell \in F$, depending on the decomposition of the normal bundle $N_{\ell /X}$.

### Definition 2.1

We say that a line $\ell \subset X$ is

- of *first type* if ,
- of *second type* if .

An equivalent geometric description is as follows: $\ell$ is of

- first type if there is a unique $\Pi _\ell =\mathbb {P}^2$ tangent to *X* along $\ell$,
- second type if there is a family $\Pi _{\ell ,t}=\mathbb {P}^2$, $t\in \mathbb {P}^1$, of 2-planes tangent to *X* along $\ell$.

Denote bythe *locus of second type lines*.

Denote by $H_F={\mathrm c}_1(\mathscr {U}^\vee _F)$ the Plücker ample line bundle on *F* and by $H_S$ the restriction on *S*. The following is a combination of [1, Lemma 1], [19, Section 3] and [14, Proposition 6.4.9].

### Theorem 2.2

If $X\subset \mathbb {P}^5$ is a cubic fourfold then *S* is 2-dimensional and is the degeneracy locus of the Gauss map, i.e., the following morphism of vector bundles:

$$\begin{aligned} {\text {Sym}}^2\mathscr {U}_F \rightarrow \mathscr {Q}_F^\vee . \end{aligned}$$

In particular ${\mathrm c}_1(K_S)=3H_S$ in ${\text {H}}^2(S,\mathbb {Q})$ and the class of *S* in $\textrm{CH}^2(F)$ is given by

$$\begin{aligned}{}[S]=5({\mathrm c}_1(\mathscr {U}_F^\vee )^2-{\mathrm c}_2(\mathscr {U}_F^\vee ))=5{\mathrm c}_2(\mathscr {Q}_F)=5\sigma _2|_F. \end{aligned}$$

If *X* is general, *S* is a smooth projective irreducible surface.

We motivate now the study of *S*. Consider the *Voisin map* of [22] $\phi :F\dashrightarrow F, \ell \mapsto \ell '$, taking a general line $\ell$ and giving the residual line $\ell '$ in the tangent 2-plane $\Pi _\ell$ to $\ell$, i.e., $\Pi _\ell \cap X=2\ell +\ell '$. Note that this is not defined on *S* nor on any lines contained in a plane contained inside *X*. Containing a plane is a divisorial condition, so for *X* outside this locus, we can resolve this map with one blowup $\widetilde{F}={\text {Bl}}_SF$ along the surface *S*. The map $\phi$ has been used in various contexts (see, e.g., [2, 21]), so it is important to understand its locus of indeterminacy. See also [14, Sections 2, 6] for further references and motivation.

As another example, [19, Theorem 0.2] proves that if *X* is very general then for every rational curve $C\in F$ of class $\beta$, the generator of ${\text {H}}_2(X,\textbf{Z})^{\textrm{alg}}$, there exists a unique $s\in S$ so that $C=\phi (q^{-1}(s))$. In [18] this is used to count the number of arithmetic genus 1 curves of fixed general *j*-invariant in *F* of class $\beta$, and in [12] to count the number of nodal rational curves of class $\beta$ respectively.

## Cohomology of $\textrm{G}(2,6)$

This section contains some ancillary computations necessary for the next section. We briefly recall the necessary notation for the Borel–Weil–Bott Theorem used to compute various cohomology groups of tautological bundles on the Grassmannian $\textrm{G}(2,6)$ with universal sub and quotient bundle $\mathscr {U},\mathscr {Q}$ respectively. For a quick introduction we found [4, Appendix A] and [17] helpful, although a more thorough reference is [23].

Denote by $\rho =(6,5,4,3,2,1)$,  respectively and $\Sigma _w$ the standard Weyl module. If $w+\rho$ is *regular*, i.e., all its components are distinct integers, then the BWB Theorem states thatis the only non-trivial cohomology group of this sheaf. In the above, $\sigma$ is the unique element of the symmetric group $S_6$ which permutes the components of $w+\rho$ so that they are non-increasing, i.e., $\sigma (w+\rho )=(\lambda _1,\ldots ,\lambda _6)$ with $\lambda _1\geqslant \cdots \geqslant \lambda _6$, and $\ell (w)$ is defined as the length of $\sigma$ in the sense of the number of transpositions of the form $(i\hspace{5pt} i+1)$ that $\sigma$ constitutes of. If on the other hand $w+\rho$ is not regular, then all cohomology groups are zero.

We recall the formula, e.g., from [7, Theorem 6.3], that if $\lambda =(\lambda _1,\ldots ,\lambda _6)$ is such that $\lambda _1\geqslant \cdots \geqslant \lambda _6\geqslant 1$ thenwhereas for an arbitrary non-increasing sequence $\lambda$, we may twist by some large weight (e.g., $(|\lambda _6|+1,\ldots ,|\lambda _6|+1)$) to make all components positive — this has the effect of tensoring the representation by a 1-dimensional one which does not change the dimension.

The first task is to decompose various tautological sheaves into irreducible representations. Here are some examples of irreducible representations

$$\begin{aligned} \Sigma _{(1,1)}\mathscr {U}^*&\cong H, \\ \Sigma _{(1,-1)}\mathscr {U}^*&\cong ({\text {Sym}}^2\mathscr {U})(H), \\ \Sigma _{(0,-1,-1,-1)}\mathscr {Q}^*&\cong \mathscr {Q}^*(1). \end{aligned}$$

### Proposition 3.1

The non-zero cohomology groups of  and  on $\textrm{G}(2,6)$ for $t=1$ arewhereas for $t=-2$ they are

### Proof

Using the following code in the SchurRing package of Macaulay2, 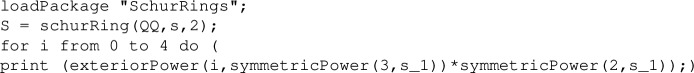 we compute the weights of the irreducible components of the representation

 as follows:

**Table:** 

*p*	$w'$	$w+\rho =(w';0,0,0,0)+\rho$	$\ell (w)$
0	(1,−1)	(7, 4, 4, 3, 2, 1)	−1
1			
		
2			
		
3			
		
4	(−5,−7)	$(1,-2,4,3,2,1)$	−1

since for example a decomposition into irreducibles for $p=2$ isIn the table, $\ell (w)=-1$ signifies that the weight *w* is not regular. From the Borel–Weil–Bott Theorem, we obtainFor $p=2$, as

$$\begin{aligned} \sigma ((6,-1,4,3,2,1)+\rho )-\rho&=(0,-1,-1,-1,-1,-2),\\ \sigma ((5,0,4,3,2,1)+\rho )-\rho&=(-1,-1,-1,-1,-1,-1) \end{aligned}$$

we obtainSimilarly, the table for  is as follows:

**Table:** 

*p*	$w'$	$w+\rho =(w'\!,0,-1,-1,-1)+\rho$	$\ell (w)$
0	(0, 0)	(6, 5, 4, 2, 1, 0)	0
1	$(0,-3)$	(6, 2, 4, 2, 1, 0)	− 1
2			
3	$(-3,-6)$	$(3,-1,4,2,1,0)$	5
4	$(-6,-6)$	$(0,-1,4,2,1,0)$	− 1

so the only non-zero cohomology groups occur for $p=0,3$. Using the same formulas as above we computeThe table for  is as follows

**Table:** 

*p*	$w'$	$w+\rho =(w'+(6,5),4,3,2,1)$	$\ell (w)$
0	$(-2,-4)$	(4, 1, 4, 3, 2, 1)	− 1
1			
			
2			
			
3			
			
4	$(-8,-10)$	$(-2,-5,4,3,2,1)$	8

givingSimilarly, the table for .

**Table:** 

*p*	$w'$	$w+\delta =(w'\!,3,2,2,2)+\rho$	$\ell (w)$
0	(0, 0)	(6, 5, 7, 5, 4, 3)	−1
1	(0,−3)	(6, 2, 7, 5, 4, 3)	5
2			
3	(−3,−6)	$(3,-1,7,5,4,3)$	−1
4	(−6,−6)	$(0,-1,7,5,4,3)$	8

giving$$\square $$

## Hodge numbers of *S*

In Theorem 2.2, we described how *S* is given as the degeneracy locus of the map

$$\begin{aligned} f:{\text {Sym}}^2\mathscr {U}_F\rightarrow \mathscr {Q}_F^\vee . \end{aligned}$$

Restricting to *S* we thus have the following sequence of vector bundles:

2 $$\begin{aligned} 0\rightarrow K\rightarrow {\text {Sym}}^2\mathscr {U}_S\xrightarrow {f|_S} \mathscr {Q}_S^\vee \rightarrow C\rightarrow 0 \end{aligned}$$

where *K* is a line bundle and *C* of rank 2. Note that there is a formula for the normal bundle of a degeneracy locus in [13, Section 3] givingThe map *f* is generically injective when considered on *F*, hence injective, and Amerik [1, Section 2] has constructed the following resolution of the ideal sheaf $I_S$ of $S\subset F$:

3 $$\begin{aligned} 0\rightarrow {\text {Sym}}^2\mathscr {U}_F(-2H)\rightarrow \mathscr {Q}^\vee _F(-2H)\rightarrow I_S\rightarrow 0. \end{aligned}$$

A short explanation is in order concerning the above. The cokernel of *f* is torsion-free by noting that the degeneracy locus *S* does not have any divisorial components (see the local computations of [8, pp. 32–33]). From this one obtains  for some line bundle *M*, and an Euler characteristic computation in [1] gives $M=2H$.

### Proposition 4.1

For *S* the surface parametrising lines of second type on a cubic fourfold *X* we have

- $$K_S^2=2835$$,
- $$\chi (\mathscr {O}_S)=450$$.

### Proof

As ${\mathrm c}_1(K_S)=3H_S \in {\text {H}}^2(S, \textbf{Q})$ and $H_S^2=315$ from Theorem 2.2, we compute that $K_S^2=2835$. To simplify notation for this proof we denote by

$$\begin{aligned} \mathscr {E}&=\mathscr {Q}_F^\vee ,\\ \mathscr {F}&={\text {Sym}}^2\mathscr {U}_F. \end{aligned}$$

To compute $\chi (\mathscr {O}_S)$ we compute first the Chern numbers of *K* and *C*. For this we use the Harris–Tu formula [13], although we follow the notation of [20]. We denote the Segre polynomialwhere ${\mathrm s}_t(\mathscr {E}), {\mathrm c}_t(\mathscr {F})$ are the Segre and Chern polynomials of $\mathscr {E}$ and $\mathscr {F}$ respectively. Written in terms of the standard Schubert cycles  on $\textrm{G}(2,6)$ we haveand in what follows we denote by . For a partition $I=(i_1,i_2,\ldots )$ we denote byso now [20, Example 5.4] (note there are some typos fixed in a later paper) gives the following intersection numbers, all taking place on *F*, i.e., intersected with [*F*] from (1):From the tangent sequence of $S\subset F$ and the fact that $K_F=0$ we obtain

$$\begin{aligned} 3H_S={\mathrm c}_1(K_S)={\mathrm c}_1(N)={}-2{\mathrm c}_1(K)+{\mathrm c}_1(C) \end{aligned}$$

from which  and hence ${\mathrm c}_2(N_{S/F})=1125$. On the other hand from the tangent sequence of $F\subset \textrm{G}(2,6)$we have ${\mathrm c}_2(T_F) = {}-3\sigma _1^2|_F+8\sigma _2|_F$, givingFrom the Noether formula we compute now

$$\begin{aligned} \chi (\mathscr {O}_S) = \frac{1}{12}\,({\mathrm c}_1(T_S)^2+{\mathrm c}_2(T_S))=450. \end{aligned}$$

$$\square $$

### Remark 4.2

Using the fact that *S* is isomorphic to $S'$ a section of the vector bundle  on $\pi :\mathbb {P}({\text {Sym}}^2 \mathscr {U}_F)\rightarrow F$, we have from [10, p. 54] the formulawhich can also be used to compute $\chi (\mathscr {O}_S)$. In fact, recently Huybrechts [14, Proposition 6.4.9] has studied the ideal sheaf $I_{S'}$, proving that sequence (2) on *S* is4for a line bundle *L* satisfying $-2L=2H_S$. From this one can, by taking Euler characteristics, also obtain that $\chi (\mathscr {O}_S)=450$. Studying cohomological vanishing on $\mathbb {P}({\text {Sym}}^2\mathscr {U}_F)$ he also obtains $h^1(S, \mathscr {O}_S)=0$ like we do in what follows.

Our aim now is to compute $q=h^1(S,\mathscr {O}_S)$ or $p_g$, noting that

$$\begin{aligned} \chi (S, \mathscr {O}_S)=1-q+p_g \end{aligned}$$

so one determines the other from the above computation. This will be achieved by computing cohomology from sequence (3). As *F* is the vanishing of a section of ${\text {Sym}}^3\mathscr {U}^\vee$, we can consider the Koszul resolution5from which it becomes clear that in order to compute groups such as

$$\begin{aligned} {\text {H}}^{\,i}(F, {\text {Sym}}^2\mathscr {U}_F(H)) \end{aligned}$$

we will need to compute the groupswhich was achieved using the Borel–Weil–Bott Theorem in Sect. 3.

### Theorem 4.3

The Hodge numbers of *S* are as follows:

$$\begin{aligned} h^{1,0}&=q=0, \\ h^{2,0}&=p_g=449,\\ h^{1,1}&=1665. \end{aligned}$$

Also, ${\text {Pic}}S \cong \textrm{NS}(S)$ and ${\text {Pic}}^\tau S\ne 0$, i.e., *S* has torsion in the Néron–Severi group and has non-trivial fundamental group.

### Proof

Tensoring sequence (5) with ${\text {Sym}}^2\mathscr {U}(tH)$ and $\mathscr {Q}^\vee (tH)$ and using the hypercohomology spectral sequence [15, B.1.5], we obtain the following second quadrant spectral sequences:From Proposition 3.1 for $t=-2$ and the first spectral sequence, we have that

$$\begin{aligned} d_{-4,8}:E_1^{-4,8}\rightarrow E_1^{-3,8} \end{aligned}$$

is the only non-trivial differential between the only two non-trivial terms of the $E_1$-page. Since ${\text {H}}^5(F, ({\text {Sym}}^2\mathscr {U}_F)(-2\,H))=0$ as $\dim F=4$, it must be that $E_\infty ^{-3,8}=0$ and so that $d_{-4,8}$ is surjective. This gives that $E_\infty ^{-4,8}=E_2^{-4,8}\cong \textbf{C}^{1008}$ and hence that ${\text {H}}^4(({\text {Sym}}^2\mathscr {U}_F)(-2H))=\textbf{C}^{1008}$ is the only non-zero cohomology group of this sheaf. Similarly, the second spectral sequence gives that

$$\begin{aligned} {\text {H}}^{\,i}(F, \mathscr {Q}_F^\vee (-2H)) = {\left\{ \begin{array}{ll} \,\textbf{C}^{561}, &{}\text {if}\;\; i=4,\\ \,0, &{}\text {otherwise}. \end{array}\right. } \end{aligned}$$

From sequence (3) we obtain now immediately that

$$\begin{aligned} {\text {H}}^{\,i}(F, I_S)=0\quad \text {for}\;\; i\leqslant 2. \end{aligned}$$

The sequence

$$\begin{aligned} 0\rightarrow I_S\rightarrow \mathscr {O}_F\rightarrow \mathscr {O}_S\rightarrow 0 \end{aligned}$$

and the fact that $h^i(F,\mathscr {O}_F)$ is 1, 0, 1, 0, 1 for $i=0,\ldots ,4$ respectively give that $h^1(S,\mathscr {O}_S)=0$. From $450=\chi (S,\mathscr {O}_S)=1-q+p_g$ we immediately obtain $p_g=449$. As $h^{1,0}=h^{0,1}=0$, so are the Betti numbers $b_1=b_3=0$. Since *S* is connected, $b_0=b_4=1$. Note that $\chi _{\textrm{top}}={\mathrm c}_2(T_S)=\sum (-1)^ib_i=2565$, giving that $b_2=2563$ and hence from the Hodge decomposition and Hodge duality that $h^{1,1}=b_2-2h^{2,0}=1665$.

For $t=1$, the first spectral sequence and Proposition 3.1 give $E_\infty ^{-2,4}=E_1^{-2,4}\cong \textbf{C}^{36}$ as the only non-zero term. Hence

$$\begin{aligned} h^2(F, ({\text {Sym}}^2\mathscr {U})(H))=36 \end{aligned}$$

is the only non-zero cohomology group of this sheaf. The second spectral sequence for $t=1$ gives that

$$\begin{aligned} h^0(F, \mathscr {Q}_F^\vee (H))=20,\quad h^2(F, \mathscr {Q}_F^\vee (H))=1 \end{aligned}$$

are the only two non-trivial cohomology groups.

The resolution of the ideal sheaf twisted by 3*H*

$$\begin{aligned} 0\rightarrow ({\text {Sym}}^2\mathscr {U}_F)(H)\rightarrow \mathscr {Q}_F^\vee (H)\rightarrow I_S(3H)\rightarrow 0 \end{aligned}$$

and the computations above give that $h^3(F, I_S(3H))=0$. Kodaira vanishing gives $h^i(F, \mathscr {O}_S(3H))=0$ for all $i\geqslant 1$ so the sequence

$$\begin{aligned} 0\rightarrow I_S(3H)\rightarrow \mathscr {O}_F(3H)\rightarrow \mathscr {O}_S(3H)\rightarrow 0 \end{aligned}$$

induces $h^2(S, \mathscr {O}_S(3H))=h^3(F, I_S(3H))$. If $K_S$ and $3\,H$ were linearly equivalent and not just equal in the group ${\text {H}}^2(S,\textbf{Q})$, then $1=h^2(S, K_S)=h^3(F, I_S(3H))$ which is a contradiction to the computation above giving $h^3(F, I_S(3H))=0$.

Since $q=h^1(S,\mathscr {O}_S)=0$ is the tangent space to the abelian variety ${\text {Pic}}^0 S$, this must be zero, giving ${\text {Pic}}S= \textrm{NS}(S)$. Since $3\,H$ and $K_S$ are cohomologically but not linearly equivalent, there must be torsion in cohomology, or in other words ${\text {Pic}}^\tau S\ne 0$.$\square$

### Remark 4.4

In [14, Remark 6.4.10], it is shown that there is a degree 2 étale cover of *S* trivialising the above torsion element, which is, from (4), the difference $K_S-3H_S\in {\text {Pic}}S$. This cover can be realised as the surface in $\mathbb {P}(\mathscr {U}_S)$ parametrising the two distinct ramification points of the Gauss map when restricted to a line.

## The surface *V* and its invariants

Let $X\subset \mathbb {P}^5$ be a smooth cubic, and denote by  the surface of triple lines, i.e., lines $\ell \subset X$ so that there exists a 2-plane so that $X\cap \mathbb {P}^2=3\ell$. Denote also by $\widetilde{V}\subset \textrm{Bl}_{S}F$, the strict transform of *V*. In [12, 4.3–4.4] we prove that if *X* is general, then *V* is an irreducible surface and $\widetilde{V}$ is its smooth normalisation, and we prove that the class of *V* in the cohomology of *F* is $21\textrm{c}_2(\mathscr {U}_F)$. In this section we will give a different geometric interpretation of $\widetilde{V}$ than the one in [12] and use this to compute the class of *V* again and some of the invariants of $\widetilde{V}$. After setting up the geometric construction, we will perform the computations using Macaulay2 as they are similar to the ones in previous sections.

We will need the following construction, suggested to us by Kuznetsov. Let  be the 11-dimensional Flag variety parametrising tuples  so that $\ell \subset \Pi$, and let $\mathscr {U}_2\subset \mathscr {U}_3$ be the universal bundles on $\textrm{Fl}$ and *L* the kernel of the surjection $\mathscr {U}_3^\vee \rightarrow \mathscr {U}_2^\vee$. Denote by *E* the rank 9 quotient of the following natural inclusion:

6 $$\begin{aligned} 0\rightarrow 3L\rightarrow {\text {Sym}}^3\mathscr {U}_3^\vee \rightarrow E\rightarrow 0, \end{aligned}$$

which is a vector bundle as the inclusion of 3*L* is of full rank at every point. The equation of the cubic *X* induces a section $t:\mathscr {O}_{\textrm{Fl}}\rightarrow {\text {Sym}}^3\mathscr {U}_3^\vee$, and hence a section $s:\mathscr {O}_{\textrm{Fl}}\rightarrow E$. Denote by $V(s)\subset \textrm{Fl}$ the vanishing locus of this section. Note that

7 $$\begin{aligned} {\text {H}}^0(\textrm{Fl},{\text {Sym}}^3\mathscr {U}_3^\vee )={\text {H}}^0(\textrm{G}(3,6),{\text {Sym}}^3\mathscr {U}_3^\vee )={\text {H}}^0(\mathbb {P}(\mathscr {U}_3), \mathscr {O}_{\mathbb {P}(\mathscr {U}_3)}(3)) \end{aligned}$$

by the usual Leray argument (in the latter two groups $\mathscr {U}_3$ is now considered as the universal bundle on $\textrm{G}(3,6)$), and these vector spaces also agree with the 56-dimensional ${\text {H}}^0(\mathbb {P}^5, \mathscr {O}_{\mathbb {P}^5}(3))$ since the pullback of $\mathscr {O}_{\mathbb {P}^5}(1)$ to the universal family $\mathbb {P}(\mathscr {U}_3)$ is $\mathscr {O}_{\mathbb {P}(\mathscr {U}_3)}(1)$. As ${\text {Sym}}^3\mathscr {U}_3^\vee$ is globally generated, so is *E*, so *V*(*s*) has dimension 2 and a general section of $\mathscr {O}_{\mathbb {P}^5}(3)$ induces a section of *E* whose zero locus is generically reduced (see [6, Lemma 5.2]).

Note that the set $V(s)\subset \textrm{Fl}$ consists of pairs $(\ell ,\Pi )$ so that $X\cap \Pi =3\ell$ or $\Pi \subset X$. To see this, note that if $(\ell ,\Pi )$ is already a zero of *t* then the equation of *X* vanishes on $\Pi$ from equation (7). For the remaining zeros of *s*, note that *L* parametrises linear forms on $\mathscr {U}_3$ which vanish on $\mathscr {U}_2$, so that from sequence (6) such a point is an $(\ell ,\Pi )$ so that $X\cap \Pi =3\ell$.

If *X* is a general cubic, then *S* is smooth and the blowup of *F* at *S* parametrises planes tangent to lines in *X* as it is known (see [14, Remark 2.2.19]) that it is isomorphic to the incidence varietyUnder the genericity assumption, *X* does not contain any $\mathbb {P}^2$’s and *V*(*s*) is necessarily reduced, so the discussion above gives.

### Proposition 5.1

If *X* is a general cubic, then *V*(*s*) is isomorphic to $\widetilde{V}$.

We give now another proof of the following fact, using the above construction, that was obtained by a different geometric construction in [12, Theorem 4.7].

### Lemma 5.2

The class of *V* in the cohomology of *F* is given by

$$\begin{aligned}{}[V]=21\textrm{c}_2(\mathscr {U}_F). \end{aligned}$$

### Proof

This can be obtained as a consequence of the construction of Proposition 5.1, and as it involves Schubert calculus computations very similar to the ones of sections above, we perform it directly in Macaulay2 in the following code, which sets up $E, \mathscr {U}_2, \mathscr {U}_3$ etc, computes the class of *V*(*s*) in $\textrm{Fl}$ as the top Chern class of *E*, pushes it forward to the Grassmannian $\textrm{G}(2,6)$, and then compares it with $21\textrm{c}_2(\mathscr {U}_F)$:
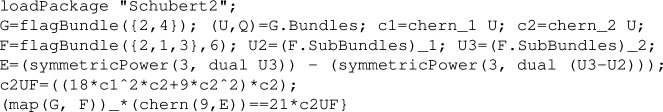
$$\square $$

Note that as *V*(*s*) is the vanishing of a section of the vector bundle *E*, its ideal sheaf has a Koszul resolutionComputing using Grothendieck–Riemann–Roch and Schubert calculus we obtain that

$$\begin{aligned} \chi (\mathscr {O}_{\tilde{V}})=1071, \end{aligned}$$

e.g., via the following Macaulay2 code 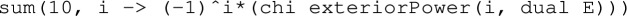 On the other hand, as the normal bundle of $\widetilde{V}$ in $\textrm{Fl}$ is given by $E|_{\tilde{V}}$ (as $\widetilde{V}$ and *V*(*s*) are isomorphic), we can compute that $K_{\tilde{V}}=3H$, for *H* the pullback of the Plücker polarisation restricted to $V\subset F\subset \textrm{G}(2,6)$, using 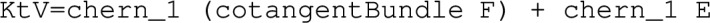 which we also computed differently in [12, Proposition 4.6] by expressing $\widetilde{V}$ as a section of a rank two bundle in ${\text {Bl}}_S(F)$. We can now easily compute $K_{\tilde{V}}^2=8505$ as follows 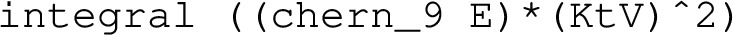


What remains in terms of the invariants of $\widetilde{V}\cong V(s)$ are the geometric genus $p_g$ and the irregularity *q*, which satisfy $p_g-q=1070$. As *E* involves indecomposable bundles on the Flag variety, the Borel–Weil–Bott computations necessary to compute either of these invariants is much more involved. Nevertheless, very recently, Mboro [16] computed that $p_g=1070$ and $q=0$ by computing the Hodge numbers of the Fano scheme of 2-planes in the cyclic cover cubic 5-fold associated to *X* and proving this is an étale 3-1 cover of $\widetilde{V}$, so all the Hodge number of $\widetilde{V}$ are now also known.

## A bound on the degree of irrationality of *F*

We recently proved in [11] that if $Y\subset \mathbb {P}^4$ is a smooth cubic threefold and *F*(*Y*) its Fano surface of lines, then the *degree of irrationality* $\textrm{irr}\hspace{0.55542pt}(F(Y))$, i.e., the minimal degree of a dominant rational map $F(Y)\dashrightarrow \mathbb {P}^2$, satisfies

$$\begin{aligned} \textrm{irr}\hspace{0.55542pt}(F(Y))\leqslant 6, \end{aligned}$$

with equality if *Y* is general. In this section we extend the construction of a degree 6 map to the Fano scheme of lines of any smooth cubic hypersurface. Whether this upper bound is optimal for a general hypersurface remains to be proven.

We recall first the construction in the case of threefolds, and elaborate on the linear system it is induced by.

### Lemma 6.1

Let $Y\subset \mathbb {P}^4$ be a smooth cubic threefold and  its Fano surface of lines. For any hyperplane $H\subset \mathbb {P}^4$ there is a degree 6 rational map

$$\begin{aligned} \phi :F\dashrightarrow Y\cap H \end{aligned}$$

which is the restriction of the rational map $\psi :\mathbb {P}\dashrightarrow H$ given by the sublinear-system $V\subset |\mathscr {O}_\mathbb {P}(1)|$ of sections corresponding to Schubert cycles $\sigma _1(\Lambda )$ for $\Lambda$ a hyperplane in *H*.

### Proof

The map $\psi _{{\text {G}}(2,5)}:{\text {G}}(2,5)\dashrightarrow H$ takes $[\ell ]$ and gives $\ell \cap H\in \mathbb {P}^4$. Consider now a $\Lambda \in |\mathscr {O}_H(1)|$. Its pullback $\psi _{{\text {G}}(2,5)}^*\Lambda$, which corresponds to lines meeting $\Lambda$, is of class $\sigma _1$ and so a section of the Plücker line bundle $\mathscr {O}_{{\text {G}}(2,5)}(1)$. Observe that this section contains all lines contained inside *H*. In other words, if

$$\begin{aligned} V=|\psi _{{\text {G}}(2,5)}^*\mathscr {O}_H(1)|\subset |\mathscr {O}_{{\text {G}}(2,5)}(1)|, \end{aligned}$$

then the base locus $\textrm{Bs}(V)$ is equal to ${\text {G}}(2,H)$. Projecting now from the $\mathbb {P}^5$ which is the span of ${\text {G}}(2,H)$ in $\mathbb {P}$ onto $\mathbb {P}^3$ we obtain the map $\psi$ whose restriction to ${\text {G}}(2,5)$ is $\psi _{{\text {G}}(2,5)}$. The map $\phi$ has degree 6 as there are 6 lines through a general point of *Y*.$\square$

### Remark 6.2

In particular, $\psi$ is the projection from the $\mathbb {P}^5\subset \mathbb {P}$ containing the Plücker embedding of ${\text {G}}(2,H)={\text {G}}(2,4)$.

### Proposition 6.3

Let $X\subset \mathbb {P}^{\,n+1}$ be a smooth cubic hypersurface for $n\geqslant 3$ and $F=F(X)\subset {\text {G}}(2,n+2)$ its Fano scheme of lines. Then

$$\begin{aligned} \textrm{irr}\hspace{0.55542pt}(F)\leqslant 6. \end{aligned}$$

More precisely, we have a degree 6 rational mapwhere $Y=X\cap H$, for $H=\mathbb {P}^{\,n}$, is a hyperplane section of *X* with one node and hence rational and $R\cong \mathbb {P}^{\,n-3}\subset \mathbb {P}^{\,n+1}$ is general. The map $\phi$ is the restriction of the mapwhere $\beta$ is given by the $n+1$ sections of $\mathscr {O}_{\mathbb {P}}(1)$ cutting out the projective space  containing the Plücker embedding of ${\text {G}}(2, H)$ and $\alpha$ is given by the space of sections of $\mathscr {O}_{\mathbb {P}}(1)$ which correspond to Schubert cycles

$$\begin{aligned} \sigma _1(T) = \{\ell \in {\text {G}}(2,n+2) \,{:}\, \ell \cap \langle T,\Pi \rangle \ne \varnothing \} \end{aligned}$$

for some fixed $\Pi \cong \mathbb {P}^2$ and *T* runs over all hyperplanes in *R*.

### Proof

Let $Y=X\cap H$ be a hyperplane section with exactly one node. Note that by projecting from the node inside $H=\mathbb {P}^{\,n}$, we obtain a birational map $Y\dashrightarrow \mathbb {P}^{\,n-1}$.

Fix now $R=\mathbb {P}^{\,n-3}$ and $\Pi =\mathbb {P}^2$ general inside $\mathbb {P}^{\,n+1}$. We will construct a degree 6 map . Consider a general point $[\ell ]\in F$. For the following two points:

$$\begin{aligned} p_\ell= & {} R\cap \langle \ell , \Pi \rangle ,\\ q_\ell= & {} \ell \cap Y, \end{aligned}$$

define now $\phi ([\ell ])=(p_\ell ,q_\ell )$. For any $q\in X$, there is a subvariety $F_q\subset F$ of dimension $n-3$ parametrising lines $[\ell ]\in F$ so that $\ell$ passes through *q*. This variety $F_q$ in fact embeds in the original $\mathbb {P}^{\,n+1}$ as a complete intersection of type (1, 1, 2, 3). Fix a $(p,q)\in \phi (F)$. The lines through *q* are parametrised by the space $F_q$ we just described. Note now that, the points $[\ell ]\in F_q$ so that $p=R\cap \langle \ell ,\Pi \rangle$ are precisely the six points of the intersection $\langle p, q, \Pi \rangle \cap F_q$. In other words $\phi$ has degree six and we can compose with a birational map  to obtain a degree six map $F\dashrightarrow \mathbb {P}^{2(n-2)}$. $\square$
